# Effects of a 3-month composite music-based recreational program on mood and executive function in institutionalized patients with advanced Parkinson’s disease

**DOI:** 10.3389/fneur.2026.1821366

**Published:** 2026-05-19

**Authors:** Haruna Aoki, Naho Shuto, Tomoyuki Hatsuse, Tatsunori Azuma, Akiyoshi Yamamoto, Kensuke Ikenaka

**Affiliations:** 1 Shizuku Works, Toyonaka, Japan; 2 Super Court Co., Ltd., Osaka, Japan; 3 Shion Hospital, Medical Corporation Yoshikenkai, Osaka, Japan; 4 Department of Neurology, Graduate School of Medicine, The University of Osaka, Suita, Japan

**Keywords:** advanced Parkinson’s disease, executive function, institutionalized patients, mood, music therapy, rehabilitation

## Abstract

Neuropsychiatric symptoms and executive dysfunction are highly prevalent in advanced Parkinson’s disease (PD), particularly among institutionalized patients, yet evidence for structured rehabilitation in this population remains limited. We conducted a 3-month longitudinal pilot study in nine institutionalized patients with clinically established idiopathic PD according to Movement Disorder Society criteria. Participants received a weekly 45-min composite music-based recreational program incorporating stretching, perioral and pharyngeal exercises, and singing with coordinated movements. Mood was assessed using the Profile of Mood States 2 (POMS-2), and executive function was evaluated using the Frontal Assessment Battery (FAB) at baseline and post-intervention. Significant improvements were observed in POMS-2 subdomains of anger–hostility (*p* = 0.014), while Total Mood Disturbance and other subdomains showed a trend toward improvement. Executive function did not significantly improve at the group level (*p* = 0.240); however, change in FAB was significantly correlated with change in Total Mood Disturbance (*r* = 0.677, *p* = 0.023). These findings suggest that a feasible music-based recreational program may modulate negative mood states in institutionalized patients with advanced PD and highlight a potential relationship between affective and executive changes warranting further investigation.

## Introduction

Parkinson’s disease (PD) is a progressive neurodegenerative disorder characterized by motor disability and a broad range of non-motor symptoms. As life expectancy increases and disease-modifying therapies remain unavailable, an increasing number of patients are surviving into advanced and late stages of PD, often requiring institutional care. In these stages, patients frequently experience severe motor impairment, neuropsychiatric complications, cognitive decline, and substantial dependency in activities of daily living (ADL).

Rehabilitation strategies, including music-based interventions, have demonstrated benefits in patients with mild-to-moderate PD (1–4). However, most interventional studies exclude patients with advanced disability or those residing in long-term care facilities. Consequently, evidence supporting structured rehabilitation in institutionalized, late-stage PD populations remains extremely limited. This evidence gap is clinically important. Advanced PD is associated with increased behavioral disturbances, affective instability, fatigue, and reduced engagement in daily activities (5–7). These symptoms not only diminish the patient’s quality of life but also increase caregiver burden and healthcare costs. In the context of rapid population aging and prolonged PD survival, addressing the needs of late-stage, institutionalized patients has become a major challenge in neurological practice.

Current international PD guidelines emphasize multidisciplinary rehabilitation (8, 9). However, most recommendations are extrapolated from studies conducted in ambulatory or moderately affected individuals. There is a striking paucity of prospective longitudinal studies evaluating structured, feasible interventions specifically designed for institutionalized patients with advanced PD. While our previous studies have primarily addressed motor and biological aspects of Parkinson’s disease (10–12), in addition, it was observed that the unmet needs of advanced-stage institutionalized patients remain insufficiently explored. Non-pharmacological approaches are particularly relevant in this population, where polypharmacy, frailty, and comorbidities limit medication adjustments. Music-based interventions are attractive because they are low-cost, safe, and easily implementable in group settings. Music engagement activates dopaminergic reward pathways and has been shown to improve emotional well-being in PD (13, 14). Nevertheless, longitudinal evidence in institutionalized advanced PD patients remains scarce. Given the growing number of patients reaching advanced, institutionalized stages, developing practical interventions that can enhance affective stability and potentially support ADL and QOL is of high clinical importance. We therefore conducted a 3-month longitudinal pilot study to evaluate the effects of a structured composite music-based recreational program on mood and executive function in institutionalized patients with advanced PD.

## Methods

### Participants

Nine institutionalized patients with clinically established idiopathic PD according to the Movement Disorder Society (MDS) clinical diagnostic criteria (7) were enrolled. All participants had been residing in the same facility for at least 5 months prior to enrollment and were receiving standard care, including routine physical rehabilitation such as gait training and seated exercise programs. These care routines were maintained throughout the study period. The present study evaluated changes before and after the introduction of the music-based recreational program in addition to this stable background care. The median age was 82.0 years (IQR 78.5–85.0), and six participants (66.7%) were female. The median disease duration was 8.0 years (IQR 3.0–12.5). The median Hoehn and Yahr stage was 4.0 (IQR 3.0–5.0). Global cognitive function, assessed using the Mini-Mental State Examination (MMSE), had a median score of 24.0 (IQR 19.5–29.5). Executive function, assessed using the Frontal Assessment Battery (FAB), had a median score of 12.0 (IQR 5.5–14.0). None of the participants had a documented diagnosis of depression or other mood disorders at baseline. In addition, no participants were receiving antidepressants or anxiolytics during the intervention period. Medication regimens were generally stable during the study period, with no systematic changes in dopaminergic or psychotropic medications during the intervention period. Minor medication changes were noted in three participants: one patient initiated amlodipine (2.5 mg), one discontinued Goreisan (Tsumura), a traditional Japanese Kampo medicine primarily used for fluid regulation, and one initiated zonisamide (25 mg).

### Ethics statement

The protocol conformed to Helsinki Declaration principles and was approved by the Osaka University review board (approval numbers 13471 and 22311) (10, 11, 15). All participants provided written informed consent before any assessment, including the collection of demographic information such as age and sex.

### Recreational program

This intervention program was defined as “music-based” because singing served as the central component of the intervention. Participants underwent a 45-min composite music-based recreational program once weekly for 3 months (12 sessions). The overall structure of each session remained consistent across all sessions. Each session consisted of the following components:

(A) stretching (10 min), aimed at relaxing muscles involved in phonation and respiration to facilitate subsequent activities; (B) vocalization exercises (10 min), including repetitive articulation tasks (e.g., “pa–ta–ka–ra”) performed with clear and loud pronunciation to enhance basic speech and voice production; (C) reading of song lyrics (5 min), to promote attention to verbal content and prepare participants for vocal expression; (D) singing (10 min), involving one consistent theme song used across sessions and one familiar song selected for each session, both performed with emphasis on loud and clear vocalization; and (E) singing combined with coordinated movements (10 min), designed as a dual-task activity to promote cognitive–motor integration. The program used simple, widely recognized songs familiar to older adults.

This intervention program was defined as “music-based” because singing served as the central component of the intervention. The preceding elements (stretching, vocalization, and lyric reading) were structured to progressively prepare participants for singing, while the final dual-task component was designed to engage executive function. This stepwise structure was intended to facilitate efficient training by breaking down complex tasks into simpler components, which may be particularly beneficial for patients with PD who often exhibit impairments in executive and dual-task processing. The intervention sessions were conducted at a fixed time of day as part of routine facility activities, regardless of ON/OFF medication state, reflecting real-world clinical practice.

### Assessments

Mood was evaluated using Profile of Mood States 2nd Edition (POMS-2™) scores. Executive function was assessed using the FAB (16). Assessments were performed immediately before initiation and immediately after completion of the 3-month program, and were conducted during the patients’ ON state to minimize variability related to motor and non-motor fluctuations.

### Statistical analysis

Statistical analysis was performed using SPSS version 29. Given the small sample size, pre–post comparisons were assessed using the Wilcoxon signed-rank test. Data are presented as median and interquartile range (IQR). Pearson correlation was used to assess the association between the change in FAB and the change in Total Mood Disturbance.

## Results

### Composite music-based recreation improved negative mood domains relevant to institutional care

**Figure 1:**
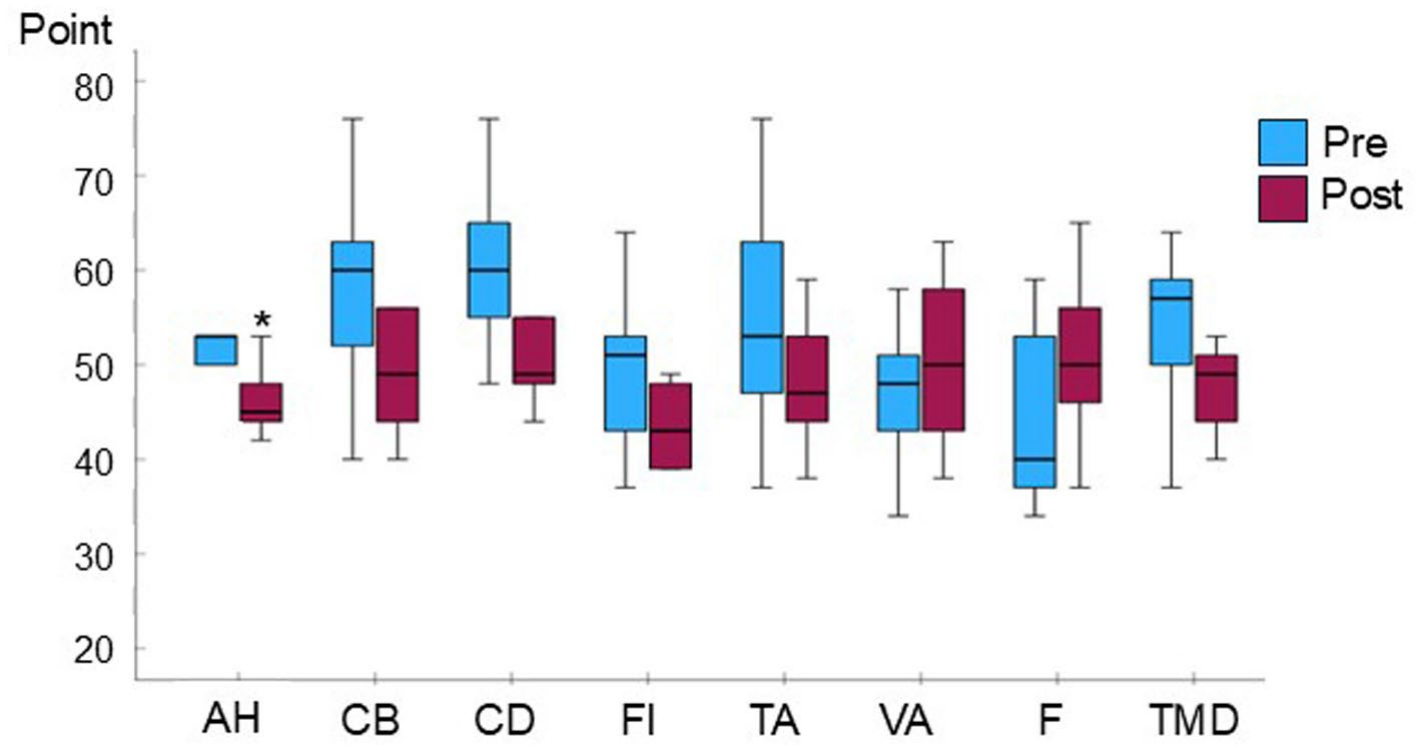
Changes in negative mood domains following a 3-month composite music-based recreational program. Box plots show POMS-2 scores at baseline (blue boxes) and after 3 months of weekly intervention (red boxes) in nine institutionalized patients with advanced Parkinson’s disease. Boxes represent interquartile ranges; horizontal lines indicate medians; whiskers denote minimum and maximum values. *n* = 9. An asterisk (*) indicates a statistically significant difference determined by Wilcoxon (*p* < 0.05). AH, anger–hostility; CB, confusion–bewilderment; DD, depression–dejection; FI, fatigue–inertia; TA, tension–anxiety; VA, vigor–activity; F, friendliness; TMD, Total ood isturbance.

Adherence to the intervention was high across participants, with a median attendance rate of 100% (IQR 79.2–100). After 3 months of weekly intervention, the anger–hostility subdomain showed a significant improvement (Table 1; Figure 1; *p* = 0.035), while other negative mood domains demonstrated consistent favorable directional changes without reaching statistical significance. Positive mood domains (vigor–activity and friendliness) showed slight increases following the intervention.

**Table 1:** Changes in POMS-2 subdomains and executive function following a 3-month music-based recreational program.

Variable	Pre-intervention	Post-intervention	Median change (Post–Pre)	*p*-value
	Median [IQR]	Median [IQR]		
AH	53.0 [46.0–58.0]	45.0 [43.0–48.5]	−8.0	0.035
CB	60.0 [52.0–63.5]	49.0 [42.0–56.0]	−11.1	0.122
DD	60.0 [51.5–68.5]	49.0 [48.0–62.5]	−11.1	0.141
FI	51.0 [42.5–55.5]	43.0 [39.0–48.0]	−8.0	0.067
TA	53.0 [44.0–64.5]	47.0 [41.0–56.0]	−6.0	0.484
VA	48.0 [42.0–52.5]	50.0 [43.0–60.5]	2.0	0.362
F	40.0 [37.0–53.5]	50.0 [41.5–57.5]	10.0	0.261
TMD	57.0 [49.5–60.0]	49.0 [43.5–52.0]	−8.0	0.106
FAB	12.0 [5.5–14.0]	12.0 [9.5–12.0]	0	0.495

*n* = 9.

AH, anger–hostility; CB, confusion–bewilderment; DD, depression–dejection; FI, fatigue–inertia; TA, tension–anxiety; VA, vigor–activity; F, friendliness; TMD, otal Mood Disturbance; FAB, Frontal Assessment Battery.

### Executive function showed a non-significant group-level change with heterogeneous individual responses

**Figure 2:**
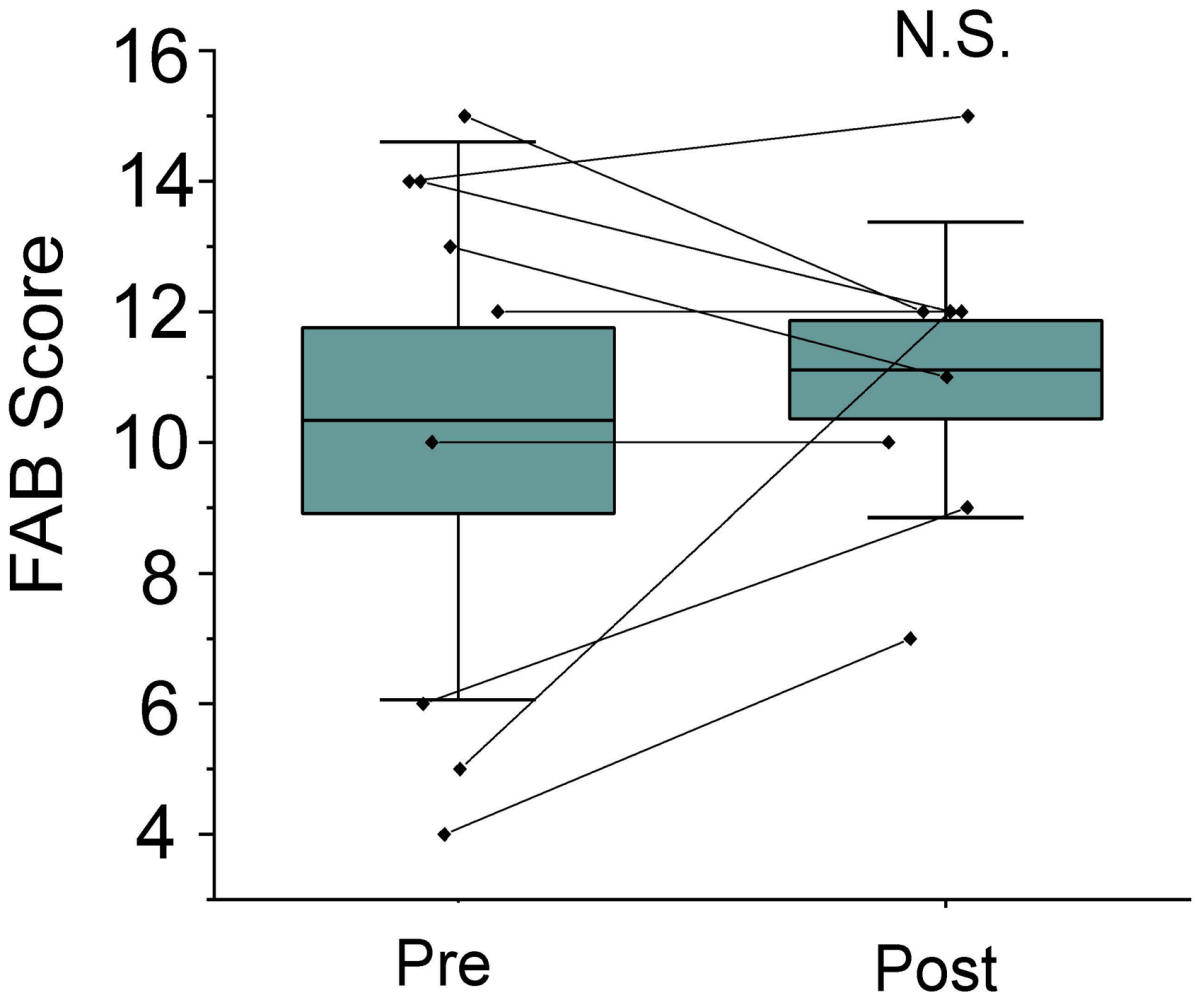
Changes in executive function following the 3-month intervention. Beeswarm plots show frontal assessment battery (FAB) scores at baseline (Pre) and after 3 months (Post). Boxes represent interquartile ranges; horizontal lines indicate medians; whiskers denote minimum and maximum values. *n* = 9*. p*-value was examined by the Wilcoxon signed-rank test.

FAB scores showed a slight increase following the program; however, this change did not reach statistical significance (Table 1; Figure 2; *p* = 0.495). At the individual level, variability in executive response was observed. Participants with lower baseline FAB scores tended to demonstrate greater improvements, whereas those with higher baseline scores showed minimal change or slight fluctuation.

### Improvement in executive function was significantly associated with a reduction in mood disturbance

**Figure 3:**
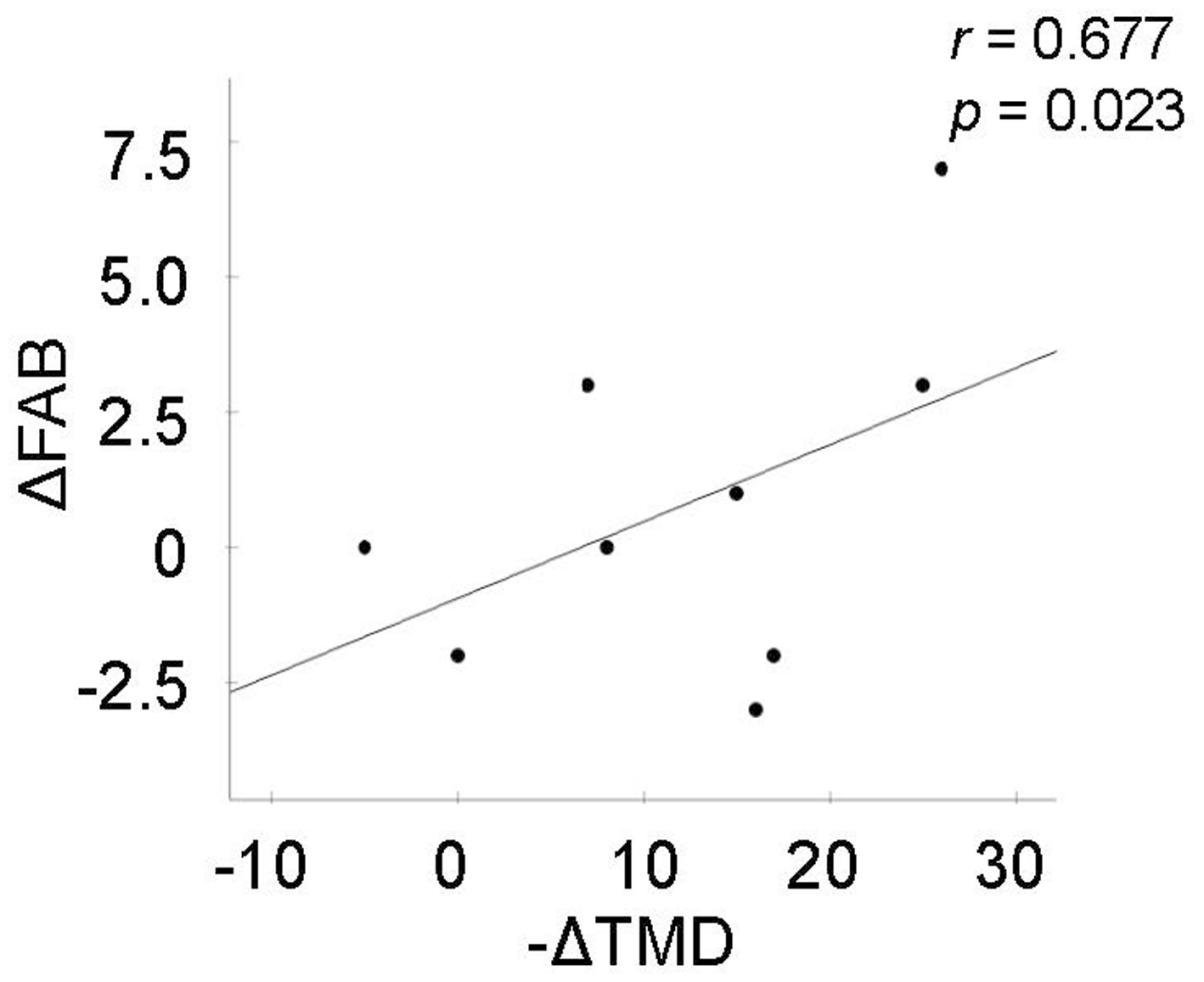
Association between changes in executive function and mood disturbance following the 3-month intervention. Scatter plot illustrating the relationship between the improvement in total mood disturbance (-ΔTMD) and improvement in FAB score (ΔFAB) over the 3-month intervention period. The solid line represents the linear regression fit. Pearson correlation coefficient (*r*) and *p*-value are shown. *n* = 9.

Interestingly, the change in FAB was significantly correlated with the change in Total Mood Disturbance of POMS-2 (Figure 3; *r* = 0.677, *p* = 0.023). Participants who experienced greater improvement in global mood disturbance tended to exhibit larger improvements in executive performance.

## Discussion

This longitudinal pilot study demonstrates that a 3-month composite music-based recreational program improved negative mood domains in institutionalized patients with advanced Parkinson’s disease. Significant improvement was observed in anger–hostility, while other negative mood domains, including confusion–bewilderment, depression–dejection, fatigue–inertia, tension–anxiety, and Total Mood Disturbance, demonstrated consistent favorable directional changes without reaching statistical significance. These findings suggest a potential benefit of the intervention despite limited statistical power. Longer intervention periods may allow for more sustained engagement and gradual consolidation of cognitive–motor and affective processes, potentially leading to more robust effects. These possibilities warrant further investigation in future controlled studies. The assessed symptoms are known to frequently complicate care in late-stage PD and contribute to reduced engagement and quality of life. Most rehabilitation studies in PD focus on ambulatory or moderately affected patients, and evidence in severely disabled, institutionalized populations remains limited. Music-based interventions have shown emotional and motor benefits in PD (1, 2) (13, 14), but longitudinal data in advanced-stage patients are scarce. Our findings suggest that even in late-stage PD, structured and feasible group-based interventions may modulate affective states. The observed effects should not be attributed solely to the musical component. The intervention included physical exercise, oropharyngeal training, and social interaction, which may have contributed to the observed improvements. Therefore, the findings likely reflect the combined effects of a multimodal intervention. The observed effects should also be interpreted in the context of ongoing standard care. Participants were already receiving routine rehabilitation and daily care activities, which were maintained throughout the study period. Therefore, the present findings reflect the additional effects of the music-based recreational program on top of a stable care environment, and it is difficult to isolate the specific contribution of the intervention alone.

Although executive function did not significantly improve at the group level, which may be partly explained by a ceiling effect in individuals with relatively preserved executive function at study entry, its significant association with mood reduction is noteworthy. Executive dysfunction in PD reflects disruption of frontal–subcortical circuits (17, 18) (15), and emotional state can influence prefrontal cognitive processing (19). The observed correlation suggests that affective stabilization may support frontal cognitive engagement in advanced PD.

Although the small sample size and imbalance in sex distribution (3 males and 6 females) precluded a meaningful analysis of sex-specific differences in response to the intervention, no apparent sex-related differences were observed; however, this finding should be interpreted with caution.

Several limitations must be acknowledged, including the small sample size and potential practice effects. The absence of a control group limits causal inference. In addition, this study was conducted in an open-label manner without blinded outcome assessment, which may have introduced detection bias. As the intervention sessions were conducted at a fixed time irrespective of ON/OFF medication state, variability related to motor and non-motor fluctuations may have influenced participants’ performance during the sessions. Although minor medication changes occurred in a small number of participants, these were limited and not systematically related to dopaminergic or psychotropic treatments. Therefore, their impact on the overall findings is considered to be minimal, although it cannot be completely excluded. Future studies should incorporate blinded evaluators and controlled designs to minimize these potential biases. Nevertheless, these preliminary findings support further controlled studies examining feasible non-pharmacological interventions in institutionalized advanced PD populations.

## Data Availability

The raw data supporting the conclusions of this article will be made available by the authors, without undue reservation.
